# Utilization of preconception care and associated factors among HIV-positive women of reproductive age attending ART clinics in government health institutions of Gamo Zone, Southern Ethiopia, 2021

**DOI:** 10.1038/s41598-026-58300-9

**Published:** 2026-06-24

**Authors:** Melkamu Tulbake, Birknesh Mereta, Eyasu Ware, Tadele Damena

**Affiliations:** 1 Department of Pediatrics and Child Health Nursing, College of Medicine and Health Sciences, Wolaita Sodo University, Wolaita Sodo, Ethiopia; 2 Department of Materinity and Reproductive Health Nursing, College of Health Sciences, Arba Minch Health Science College, Arba Minch, Ethiopia; 3 Department of Public Health, College of Health Sciences, Arba Minch Health Science College, Arba Minch, Ethiopia; 4 Department of Emergency and Critical Care Nursing, College of Medicine and Health Sciences, Jinka University, Jinka, Ethiopia

**Keywords:** Preconception care, Utilization, Human immune deficiency virus, Gamo zone, Diseases, Health care, Medical research, Risk factors

## Abstract

Preconception care is an important intervention aimed at improving maternal and neonatal outcomes among women of reproductive age and HIV-positive women. Inadequate utilization of preconception care may increase the risk of vertical HIV transmission and adverse pregnancy outcomes. To assess utilization rate of preconception care and associated factors among Human Immune Deficiency Virus positive reproductive age women attending Anti-Retroviral Treatment clinics in Gamo zone, 2021. An institution-based cross-sectional study was conducted from March 1 to April 1, 2021. A total of 559 participants were selected using a simple random sampling technique. Data were collected through a structured, pretested, interviewer-administered questionnaire and analyzed using SPSS version 25. Variables with a *p*-value < 0.25 in the bivariable analysis were included in the multivariable logistic regression model. Statistical significance was declared at a *p*-value < 0.05 with a 95% confidence interval. Total of the 559 HIV-positive women selected for the study, 579 participated, resulting in a response rate of 96.5%. overall utilization of preconception care was 32%. Education, Monthly income, History of pregnancy, Number of pregnancy and Occupation were significantly associated with utilization of preconception care with (AOR: 4.42; 95% CI 1.48–13.17), (AOR: 2.85; 95% CI 1.41–5.74), (AOR: 2.99; 95% CI 1.18–7.71), (AOR: 2.97; 95% CI 1.28–6.91), and(AOR: 0.45; 95% CI 0.21–0.96) respectively. The utilization of preconception care among HIV-positive women was low. Educational status, occupation, monthly income, history of pregnancy, and gravidity were significantly associated with preconception care utilization. Healthcare providers, health facilities, and public health programs should enhance awareness creation, counseling, and accessibility of preconception care services, particularly among women with lower socioeconomic status and limited reproductive experience.

**Supplementary Information:**

The online version contains supplementary material available at 10.1038/s41598-026-58300-9.

## Introduction

Preconception care (PCC) is the provision of biomedical, behavioral, and social health interventions to women and couples prior to conception with the goal of improving their health and reducing behaviors, environmental exposures, and individual risk factors that may contribute to poor maternal and child health outcomes^1,2^.

Preconception care offered during the months preceding conception provides an opportunity to address modifiable risk factors, promote healthy behaviors, and improve long-term maternal and child health outcomes^2,3^. Recognizing its importance, several international organizations and professional associations have recommended integrating PCC into routine reproductive and primary healthcare services. The World Health Organization (WHO) recommends that PCC address a broad range of health issues, including nutritional disorders, anemia, diabetes, tobacco use, genetic conditions, environmental health risks, infertility, sexually transmitted infections, HIV, mental health conditions, interpersonal violence, and unintended or closely spaced pregnancies^1,2^. Effective implementation of PCC contributes to healthier pregnancies, reduced maternal and neonatal complications, and improved reproductive health outcomes^2,3^.

Preconception care is particularly important for women living with HIV. Advances in antiretroviral therapy (ART) have improved life expectancy and reproductive aspirations among women with HIV, increasing the need for comprehensive reproductive health services. PCC for HIV-positive women aims to optimize maternal health before conception, prevent unintended pregnancies, reduce the risk of mother-to-child transmission (PMTCT), and minimize HIV transmission to uninfected sexual partners^4,5^. Comprehensive PCC includes counseling on reproductive intentions, safer conception methods, family planning, adherence to ART, management of comorbidities, screening and treatment of sexually transmitted infections, and education regarding pregnancy-related risks and outcomes^4,5^.

Globally, utilization of preconception care remains suboptimal, particularly in low- and middle-income countries. Studies have shown that utilization is influenced by educational status, socioeconomic conditions, reproductive history, awareness of PCC services, partner support, and accessibility of healthcare services^6–8^. Although PCC has been recognized as an essential component of maternal and reproductive healthcare, its implementation remains limited in many developing countries^2,6^.

In Ethiopia, maternal and child health improvement remains a major public health priority, and efforts have been made to strengthen (HIV) prevention and treatment services, including the integration of PMTCT services into routine maternal healthcare^9,10^. Ethiopia continues to face a substantial burden of HIV among women of reproductive age, making reproductive health counseling and preconception care important components of HIV care^9,10^. Despite the expansion of antiretroviral therapy (ART) services and prevention of mother-to-child transmission (PMTCT) programs, reproductive counseling and preconception care remain inadequately integrated into HIV care services. Consequently, many HIV-positive women do not receive appropriate preconception counseling before becoming pregnant^6,9^. Furthermore, studies examining preconception care utilization among HIV-positive women in Ethiopia remain limited and have reported varying findings^6–8^.

**Fig. 1:**
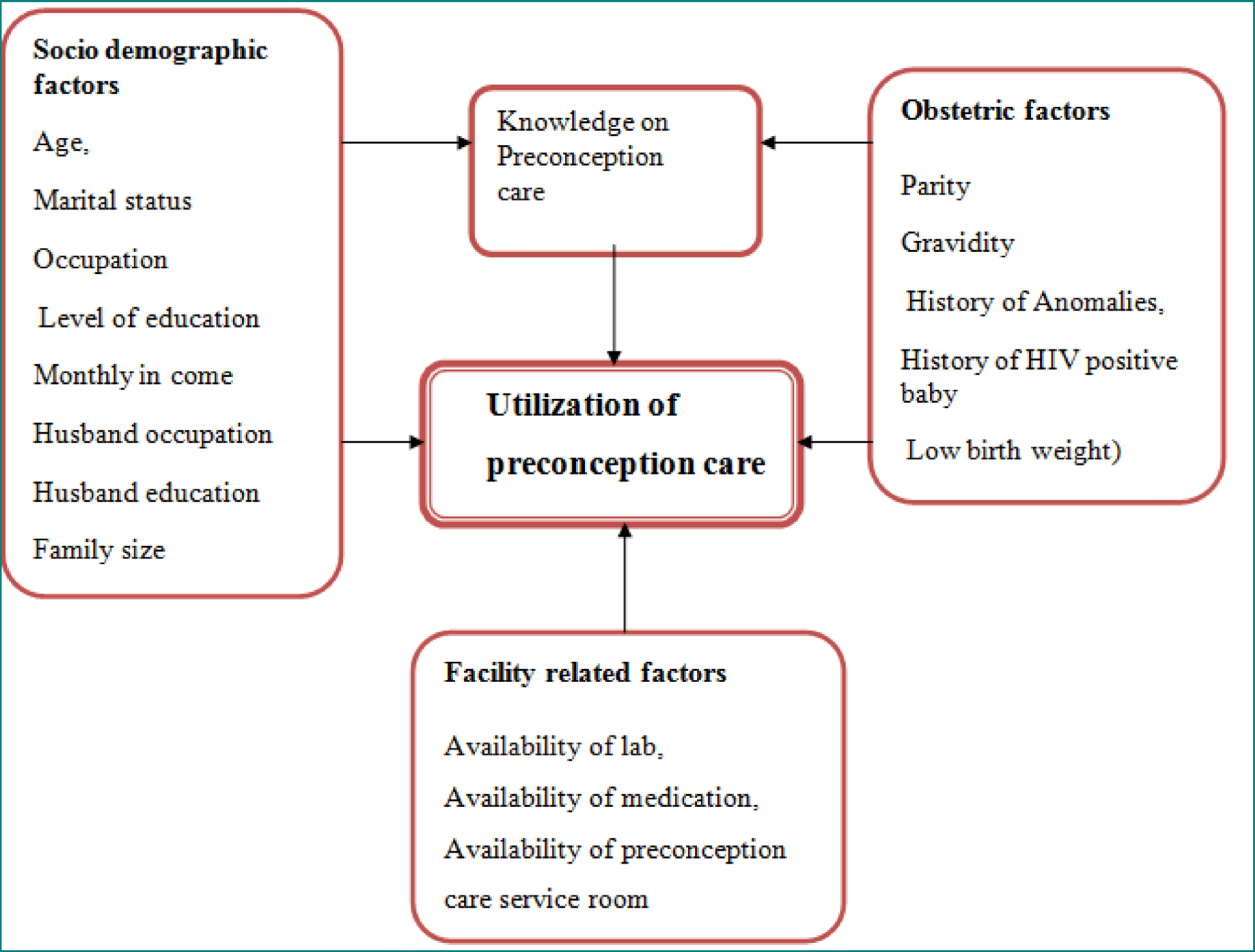
Conceptual framework for the study utilization of preconception care and associated factors among HIV positive reproductive age women in the art ART clinics.

Although several studies have assessed preconception care utilization in different settings, evidence remains scarce regarding PCC utilization and its associated factors among HIV-positive women in Southern Ethiopia, particularly in the Gamo Zone. Understanding the level of PCC utilization and identifying factors influencing its use among HIV-positive women are essential for improving reproductive health services and reducing adverse maternal and neonatal outcomes. Therefore, this study aimed to assess the utilization of preconception care and associated factors among HIV-positive women of reproductive age attending ART clinics in Gamo Zone, Southern Ethiopia (Fig. 1).

## Methods and materials

### Study area and period

The study was conducted in the Gamo zone of SNNPR. It has 14 woredas and one zonal town. The total population is 1,580,000 out of which 837,400 are females. Among this 368,140 (23.3%) females are under the reproductive age category. 3243 adults are on ART; among those 1728 are women of reproductive age. There are 4 primary hospitals and one general hospital, 57 health centers, 299 health posts, and also, private facilities are found in the zone. Three hospitals and ten health centers provide ART services in Gammo Zone.Arbaminch town is the zonal town of the Gamo zone which is located 531 km from Addis Ababa, the capital city of Ethiopia, and 227 Km from Hawasa the regional capital city of SNNPR. The study was conducted from March 1 to April 1, 2021.

### Study design

A facility-based cross-sectional study was conducted.

### Population

#### Source population

All HIV-positive women of reproductive age group attending ART clinics in theGamo zone governmental health facilities.

#### Study population

All selectedHIV-positive women of reproductive age attending ART clinics in theGamo zone government health facilities during the study period.

## Eligibility criteria

### Inclusion criteria

HIV-positive women of reproductive age attending ART clinics in the public health institution during the study period without considering marital and pregnancy status.

### Exclusion criteria

critically ill HIV-positive reproductive-age women who cannot communicate attending ART clinics in the selected public health institutions during the study period.

### Sample size determination

The sample size was determined by using the single population proportion formula. considering the following assumptions: 95% confidence interval, 5% margin of error, and best estimate of 50% prevalence of of preconception care among HIV-positive reproductive-age women because of there is no study in a similar setting to this study.

n = (Zα/2)^2^ × p (1−p)**/**d2. Where,

Zα/2 = Standard normal variable at 95% confidence level (1.96),

p = Population proportion prevalence,

d = Margin of error,

n = Sample size, so.

n = (1.96)^2^ × 0.5(1−0.5)/ (0.05)^2^.

*n* = 384.

Sample size for the second objective, for the factors associated with utilization of preconception among HIV positive reproductive-age women was calculated using EPI Info version 7.2 using a two-population proportion formula assuming a 95% confidence interval and 80% power of the study as follows:

**Table 1:** Sample size for the associated factors of utilization of preconception among HIV positive reproductive-age women Gammo Zone, 2021.

Variables	Power (%)	Ratio	Outcome in un exposed	Outcome in exposed	Sample size	5% contingency	Final sample size	References
Joint discussion and plan with partner	80	1	14.2	23.9	551	27.5 ~ 28	579	^11^

The sample size for the second specific objective is larger than that of the first, so this study used 579.

### Sampling procedure

There are three hospitals and ten health centers in Gamo Zone that provide ART services. The study population consisted of 1162 HIV-positive women of reproductive age attending ART clinics in these government health facilities during the study period. A multistage sampling technique was employed. First, all three hospitals were included using a census method, while four health centers were selected from the ten health centers using a simple random sampling technique.

After selection of the facilities, the total sample size was proportionally allocated to each selected health facility based on the average monthly number of HIV-positive women attending ART services. A sampling frame was prepared using ART clinic registration logs, and participants were selected using a simple random sampling technique (computer-generated random numbers) from the list of eligible women in each facility.

**Fig. 2:**
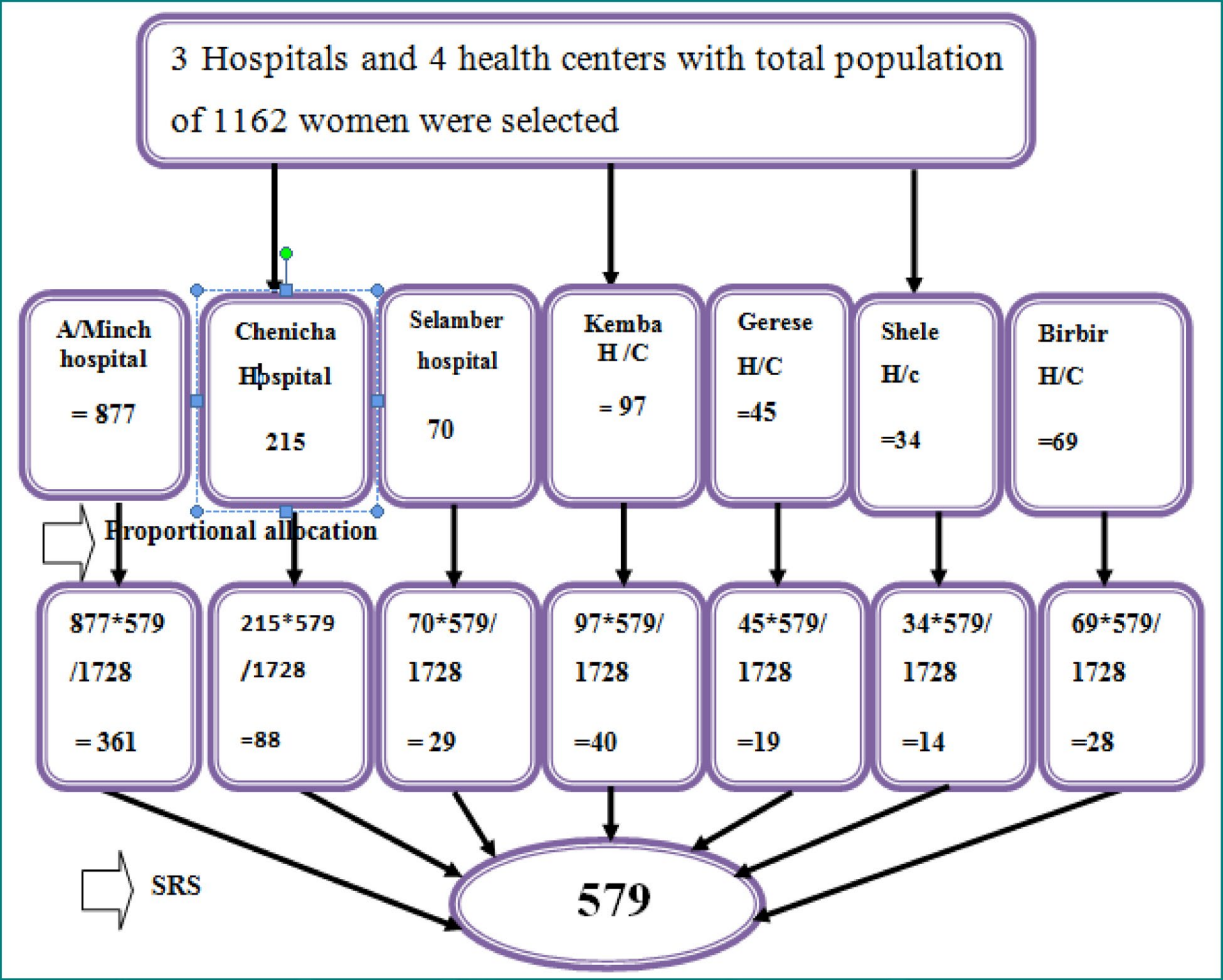
Schematic presentation of the Sampling procedure for the study utilization of preconception care and associated factors among HIV positive reproductive-age women in Gamo zone.

In cases of non-response or absence during data collection, no replacement was made, and the next randomly selected participant from the sampling frame was approached to maintain the required sample size (Fig. 2).

## Study variables

### Dependent variables

Utilization of preconception care.

### Independent variables

**Knowledge on preconception care** Information on preconception care and level of knowledge.

**Socio-demographic factors age**, marital status, educational status of the mother, occupation, and educational status of the husband, occupation of husband, monthly income, and family size.

**Obstetric factor** Parity, gravidity, history of (abortion, stillbirth, congenital anomaly, and neonatal death), history of contraceptive use.

**Facility related factors** Availability of PCC service provision room, Availability of adequate laboratory service availability of adequate medication.

### Operational definition

**Preconception care (PCC)** Preconception care refers to a set of biomedical, behavioral, and social health interventions provided to women and couples before conception to improve maternal and child health outcomes by identifying and modifying risk factors before pregnancy^1,2^. In this study, preconception care includes HIV-related interventions (male partner HIV testing, promotion of safer sexual practices, antiretroviral therapy adherence, pregnancy planning, and plasma viral load assessment), family planning services, sexually transmitted infection (STI) screening and treatment, nutritional counseling, iron and folic acid supplementation, immunization services, counseling on alcohol cessation, and counseling on cigarette smoking cessation^4–6^.

**Knowledge of preconception care** Knowledge of preconception care refers to the information and understanding that HIV-positive women of reproductive age have regarding the purpose, benefits, components, and availability of preconception care services^6,7^.

**Good knowledge** Knowledge of preconception care was assessed using structured knowledge questions. Each correct response was assigned a score of one^1^, while incorrect or “do not know” responses were assigned a score of zero (0). The total knowledge score was obtained by summing the scores of all knowledge-related questions. Respondents who scored greater than or equal to the mean knowledge score of all participants were classified as having good knowledge. This approach has been widely applied in Ethiopian studies assessing knowledge of preconception care where no standardized cut-off point exists^6–8^.

Poor Knowledge: Respondents who scored below the mean knowledge score of all participants on the preconception care knowledge assessment questions were categorized as having poor knowledge^6–8^.

**Utilization of preconception care** Utilization of preconception care refers to receiving at least one preconception care intervention, including counseling, advice, treatment, screening, preventive services, or lifestyle modification before pregnancy. In this study, a woman was considered to have utilized preconception care if she reported receiving one or more components of preconception care before becoming pregnant^4,6^.

### Data collection procedures

Data were collected using a pre-tested, structured, interviewer-administered questionnaire through face-to-face interviews. Eight trained health care providers served as data collectors, and three supervisors closely monitored the data collection process to ensure quality and consistency.

The questionnaire contains five parts including socioeconomic and demographic characteristics, obstetrics factors, Knowledge related factors, utilization, and facility-related factors of PCC.

Confidentiality of information, respondent’s rights, informed consent, and technique of interview supported with communication and discussion every day with the supervisors and data collectors about the problem faced were made during the data collection process.

### Data quality management/assurance

Various activities were performed to assure the quality of data. The English version questionnaire would be translated into Amharic by a person knowing both of the languages. The principal investigator and supervisors were frequently supervising the data collection process by checking the completeness of the required type of data to correct faults if any on the site of data collection. Proper training was givento the interviewers and supervisors of the data collection procedures, proper categorization, and coding of the questionnaire.

The quality of data was assured by properly designing and pre-testing the questionnaire, the questionnaire was pre-tested on (5% of total sample size) respondents in health institutions that had similar characteristics with the study population.

Every day, all the completed questionnaires were reviewed and checked for completeness and relevance by the supervisors and every other day by the principal investigator, and all the necessary feedback was offered to data collectors the next morning before the actual procedure.

#### Ethical approval and informed consent

All methods were carried out in accordance with relevant guidelines and regulations. The study protocol was approved by Arba Minch health science college, Approval No. Amchs/01/20/7036. Informed consent was obtained from all participants prior to participation. Participation was voluntary, confidentiality was maintained, and participants could withdraw at any time without consequence.

### Data processing and analysis

Data were entered by using Epi data version 3.1 and exported to SPSS statistical software package version 25 for data cleaning and analysis.

During analysis the variables are defined, categorized, and recoded, then frequencies and percentage of the different variables were computed. The bivariate analysis was used primarily to check which variables had an association with the dependent variable individually and multivariable logistic regression was conducted to analyze factors that have an independent association with the utilization of preconception care.

All variables were found to be associated with the main outcome variables with 95% CI in the bi-variate model i, e. *p*-Value < 0.25 and other important variables from other Studies will be a candidate for the multivariable model analysis, and variables having an independent association with outcome variable at *p*-value < 0.05 was considered as statistically significant.

Before fitting the multivariable logistic regression model, Multi-co-linearity test was carried out to see the correlation between independent variables using collinearity statistics (variance inflation factor < 4) and a model fitness test (Hosmer-Lemeshow statistic = 0.6). Potential interaction effects between key independent variables were assessed and retained in the final model if statistically significant. Data were checked for completeness before analysis, and questionnaires with substantial missing information were excluded. Variables with minimal missing data were analyzed using complete-case analysis.

## Result

### Socio-demographic characteristics of Respondents

A total of 559 reproductive-age women participated in the study, yielding a response rate of 96.5%. The age of the study participants ranged from 15 to 48 years with a mean age of 32.52 ± 7.439 SD and two hundred ninety-one (51.2%) of respondents were married. The mean monthly income was 3333.34 while 144 (31.3%) studied participants earn less than 1000 ETB. Occupations of the respondents and the rest were also mentioned in the table below (Table 1).

**Table 2:** Socio-Demographic Characteristics of Respondents for preconception care among reproductive-age women attending ART clinic at Gamo zone government health institution, South Ethiopia, 2021 (*N* = 559).

Variable		Frequency	Percent (%)
Age in year	15–24	78	14.0
	25–34	235	42.0
	35–49	246	44.0
Marital status	Married	291	52.1
	Single	79	14.1
	Divorce	99	17.7
	Widowed	90	16.1
Educational status of the respondent	no read write	118	21.1
	Read-write only	95	17.0
	Primary	135	24.2
	Secondary	139	24.9
	College and above	72	12.9
Occupation of respondent	Government employ	82	14.7
	Private employ	178	31.8
	Student	67	12.0
	Housewife	174	31.1
	NG Employ	58	10.4
Educational status of husband	No read write	43	14.8
	Read-write only	36	12.4
	Primary	69	23.7
	Secondary	70	24.1
	College and above	73	25.1
Occupation of husband	Government employ	82	28.2
	Private employ	86	29.6
	Student	14	4.8
	NG Employ	5	1.7
	Daily laborer	91	31.3
	Farmer	13	4.5
Total house hold Monthly income in ETB	<=1000	144	31.1
	1001–2500	115	25.0
	2501–5000	108	23.5
	5001–10,000	78	17.0
	> 10,000	15	3.3
Family size	< 4	363	64.9
	> 4	196	35.1

### Knowledge of respondents towards preconception care

**Fig. 3:**
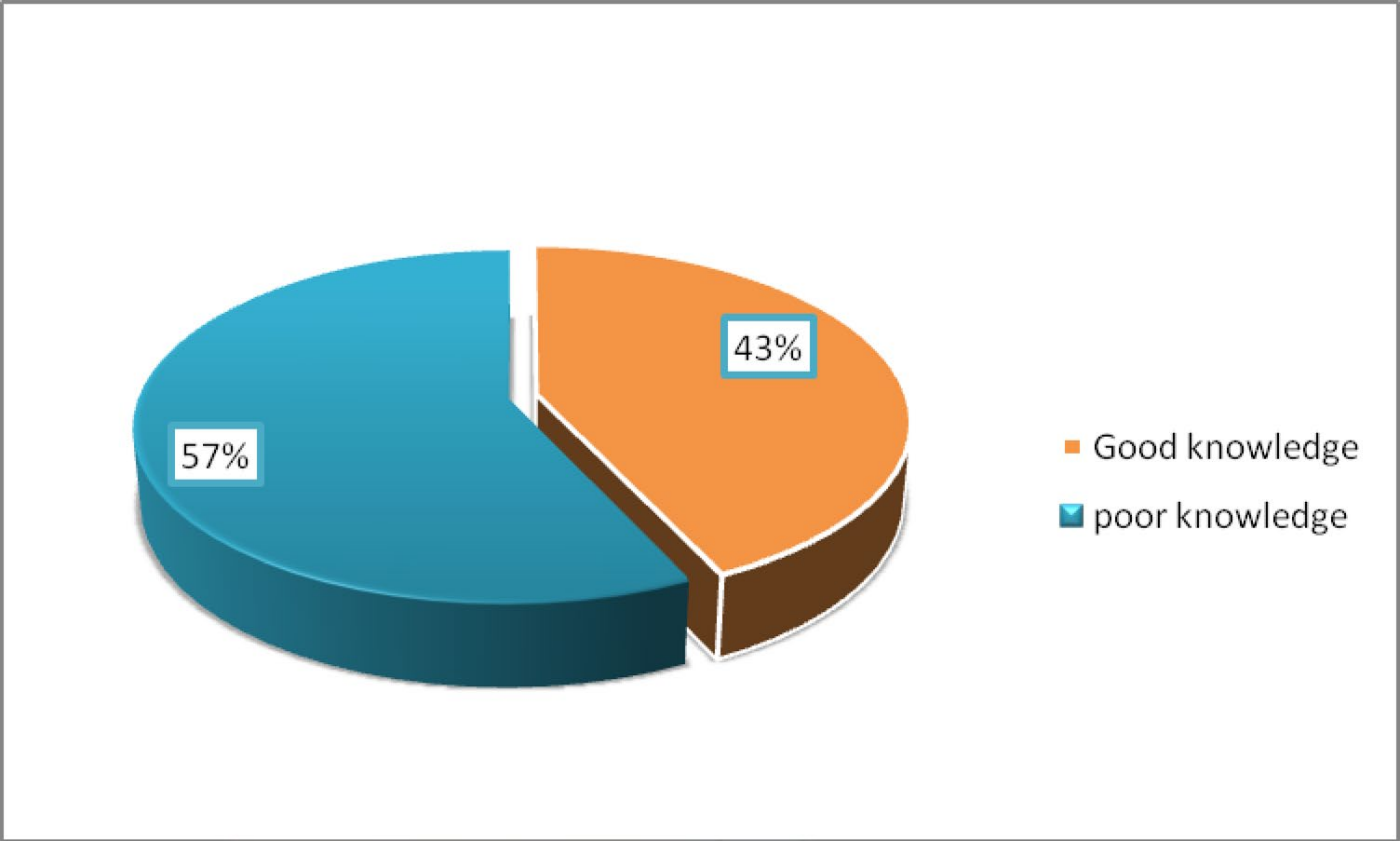
Level of knowledge among reproductive-age women attending ART clinic at Gamo zone government health institutions 2021.

Of the total of 559 participants, 286 (51.2%) of women have information about preconception care before. Of those who have information; health professionals were the major source of information 222 (75.3%) followed by media 74 (25.1%), friends 45 (15.2%), and from families 23 (7.7%). Based on the PCC component questions response 123 (43%) of women have good knowledge of PCC (Fig. 3 and Table 2).

**Table 3:** Respondents Knowledge on Preconception care among HIV positive reproductive age women attending ART clinic in Gamo zone governmental health institutions 2021.

Variables		Frequency	Percent%
Mention PCC components *N* = 286	< 5	186	65.0
	>=5	100	35.0
information on PCC *n* = 559	Yes	286	51.2
	No	273	48.8
PCC service users *N* = 286	Only male	7	2.4
	Female only	160	55.9
	For both female & male	119	41.6
PCC is important *N* = 286	Yes	279	97.6
	No	7	2.4
For whom PCC important *N* = 286	For baby, only	4	1.4
	For mother, only	112	39.2
	For all	170	59.4
Site for PCC service *N* = 286	At home	0	0.00
	At health institution	256	89.5
	At home and health institution	30	10.5

#### Respondents reason for not getting information on PCC (Fig. 4)

**Fig. 4:**
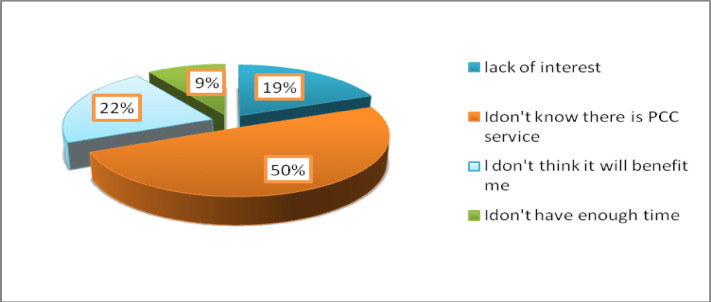
Reason of respondents for not getting information about PCC among women attending ART clinic at Gamo zone government health institutions 2021.

### Utilization of preconception care among respondents

**Fig. 5:**
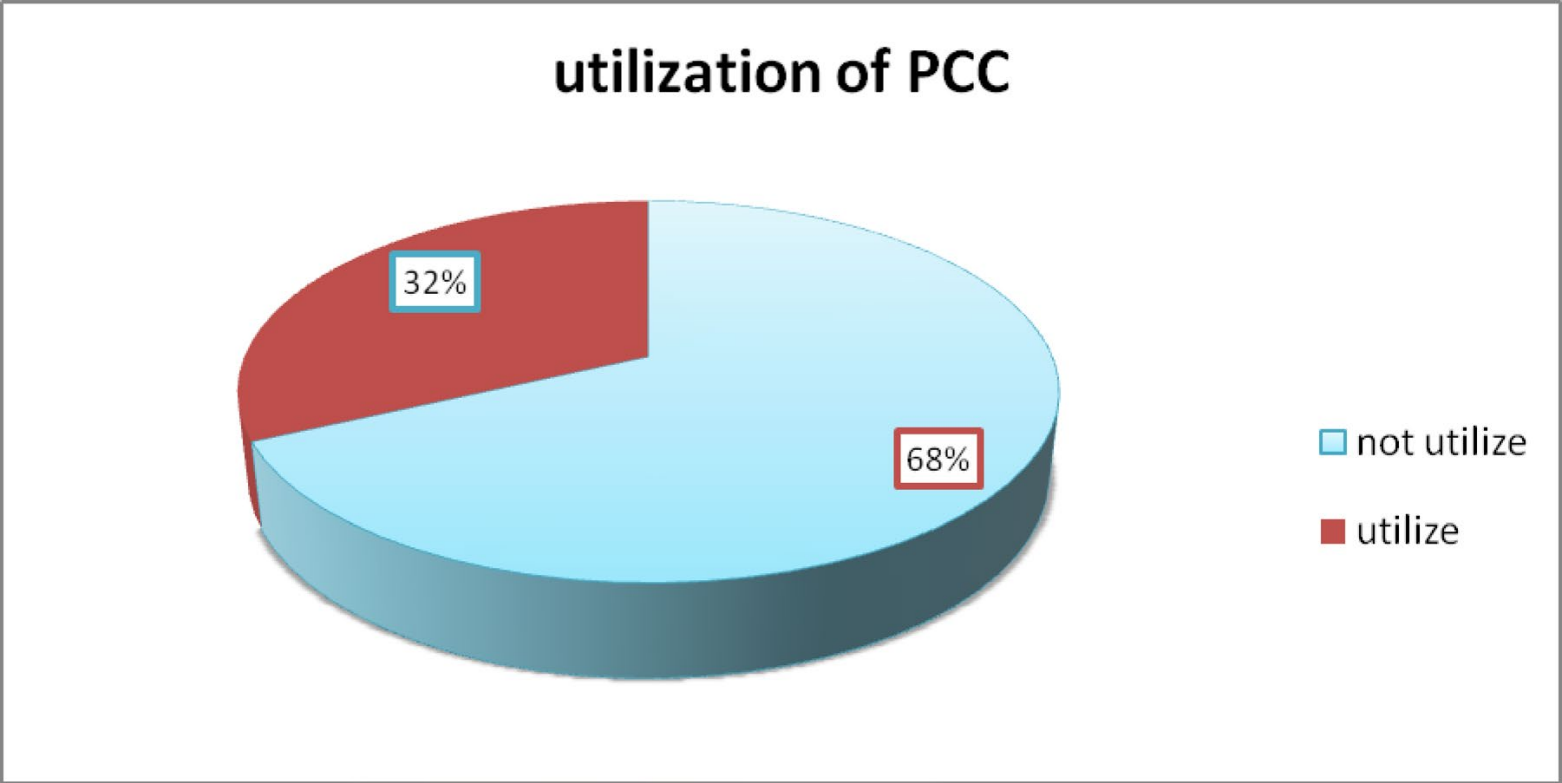
Overall utilization of preconception care among HIV positive reproductive. Age group women attending ART clinic at Gamo Zone Government health institutions 2021.

In general, as displayed in the below figure the result from the study showed that 178 (32%) of reproductive age women utilized preconception care services. Among those HIV testing and counseling and ART 559 (100%), partner counseling and testing 134 (24%), and 187 (33.5%) had a discussion about preconception care with healthcare care providers (Fig. 5 and Table 3).

**Table 4:** Frequency distribution of utilization of preconception care components among HIV-positive women in Gamo zone government health institutions, 2021.

Preconception care components	Frequency	Percent (%)
HIV testing and counseling	559	100
Anti Retroviral therapy	559	100
Partner counseling and testing	134	24
Pregnancy desire and viral load assessment	45	8.1
Nutrition counseling	71	12.7
Ferrous supplementation	23	13.1
Immunization	45	4.1
Family planning	143	25.6
Counseling on alcohol and cigarette cessation	34	6.1
Other (STI screening and treatment).	9	1.6

### Obstetrics characteristics of the participants

**Fig. 6:**
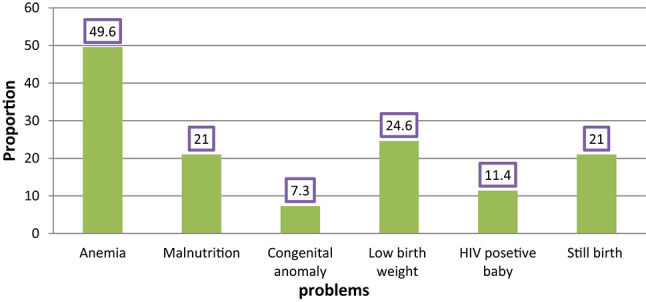
Proportion of women who experience pregnancy and birth-related complications among HIV positive reproductive age group women attending ART clinic at Gamo Zone Government health institutions 2021.

Of the total 559 participants, 452 (80.9%) respondents had a pregnancy history and 393 (86.9%) gave birth in the past. Among those majority of women 356 (78.6%) were multigravida and 261 (57.7%) of women were multiparous. 59 (13.1%) of the women experience abortion among those 44 (75%) of them were spontaneous. 44(7.9%) of women are currently pregnant. Of those 35 (79.5%) pregnancies are desired. Of the total respondents, 236 (42.3%) are wanted a child for the future. More than half 249 (44.5%) of women experienced pregnancy after they become HIV positive and 128 (51.4%) of women develop problems through their pregnancy and delivery time (Fig. 6).

### Facility related and other factors

Of the total of 178 women who had preconception care, 178 (100%) get the service from health institutions, among those 98 (55.1%) and 107 (60.1%) of women say they get laboratory services and medications respectively from health institutions.

**Fig. 7:**
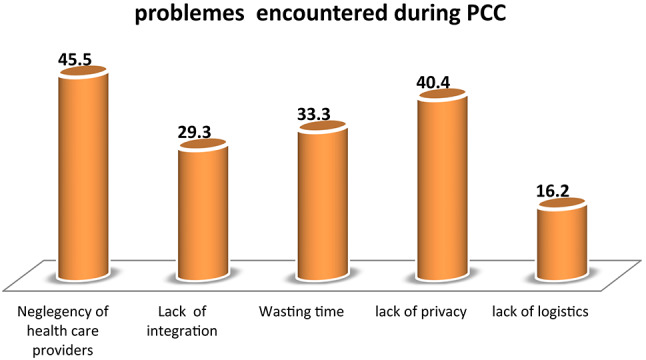
Problems encountered during utilization of preconception care among reproductive-age women attending ART clinic at Gamo zone Government health institutions 2021.

Among those 99 (55.6%) of women encountered challenges during care, and the most frequently mentioned challenge was negligence from health care providers. however, all the participants mentioned that there is no unit for PCC service in health institutions (Fig. 7).

#### Factors associated with utilization of preconception care

In bivariate analysis, from socio-demographic characteristics (age of the respondents, marital status, educational level of the respondents, occupation of respondents, education level and occupation of husband, family size, and household monthly income), from knowledge (women’s information on preconception care, knowledge of women about preconception care service), from the obstetrics characteristics (history of pregnancy, history abortion, history of birth, number of pregnancy, Desire of having child, Problem during pregnancy and delivery time, Duration of living with HIV) were the candidate variables (variables which had a *p*-value of ≤ 0.25) for multivariable logistic regression. The educational level of the respondents, occupation of the respondent, monthly income, history of pregnancy, and Number of pregnancies were statistically significant factors in multivariable logistic regression (*p* < 0.05).

Multivariable logistic regression analysis revealed that educational status, monthly income, occupation, and history of pregnancy were significantly associated with preconception care utilization. Women who were able to read and write were 4.34 times more likely to utilize preconception care compared with women who could not read and write (AOR = 4.34; 95% CI 2.00–9.66). Similarly, women with primary education (AOR = 3.43; 95% CI 1.58–7.47), secondary education (AOR = 2.38; 95% CI 1.02–5.55), and college education or above (AOR = 4.42; 95% CI 1.48–13.17) were more likely to utilize preconception care than women who could not read and write.

Monthly income was also significantly associated with preconception care utilization. Women with a monthly income of 1001–2500 ETB were 2.91 times more likely to utilize preconception care than those earning less than 1,000 ETB (AOR = 2.91; 95% CI 1.61–5.26). Likewise, women with a monthly income of 5001 ETB or more were 2.85 times more likely to utilize preconception care compared with those earning less than 1000 ETB (AOR = 2.85; 95% CI 1.41–5.74). Regarding occupation, women employed in the private sector were 55% less likely to utilize preconception care than government employees (AOR = 0.45; 95% CI 0.21–0.96).

Furthermore, women who had a history of pregnancy were approximately three times more likely to utilize preconception care than those with no history of pregnancy (AOR = 2.99; 95% CI 1.18–7.71). Women who had 2–4 history pregnancy were 2.59 times (AOR: 2.59; 95% CI 1.37, 4.93) and women who five and above history of pregnancy were 2.97times (AOR: 2.97; 95% CI 1.28, 6.91) more likely to utilize preconception care than one and no history of pregnancy (Table 4).

**Table 5:** Factors independently associated with utilization of preconception care among HIV positive reproductive-age women in Gamo Zone government health institutions, South Ethiopia, 2021.

Variables		Utilization		COR (95%) CI	Adjusted OR(95%)CI	*p*-value
		Yes	No			
Educational status of the respondent	No, read and write	13	105	1	1	1
	Only read and write	40	99	4.93 (2.42–10.02)	**2.34 (0.96–8.66)**	0.12
	Primary education	49	86	4.60 (2.34–9.04)	**2.03 (0.98–6.47)**	0.10
	Secondary education	36	59	3.26 (1.65–6.46)	**2.38 (0.82–5.7)**	0.078
	Collage and above	32	40	10.1 ( 4.82–21.17)	**4.42 (1.48–13.17)**	**0.001**
Occupation of the respondent	Government employ	45	37	1	1	1
	Private employee	45	133	0.28 (0.16–0.48)	**0.45 (0.21–0.96)**	**0.039**
	Student	7	60	0.10 (0.04–0.26)	0.542 (0.14–1.26)	0.27
	House wife	47	127	0.30 (0.18–0.53)	1.45 (0.21-1.00)	0.35
	NG employee	34	24	1.16 (0.59–2.30)	1.21 (0.53–2.75)	0.65
Monthly income in ETB	<=1000	27	126	1	1	1
	1001–2500	42	70	5.21 (3.09–8.79)	2.03 (0.97–5.26)	0.062
	2501–5000	52	63	3.79 (2.22–6.46)	1.76 (0.92–3.41)	0.07
	>=5001	52	46	7.14 (4.14–12.30)	**2.85 (1.41–5.74)**	**0.0035**
History of pregnancy	Yes	169	283	6.50 (3.20-13.21)	**2.99 (1.18–7.71)**	**0.016**
	No	9	98	1	1	1
Number of pregnancy	1	18	78	1	1	1
	2–4	29	44	4.02 (2.59–6.89)	2.01 (0.99–4.93)	0.09
	>=5	122	161	4.30(2.31–7.98)	**2.97 (1.28–6.91)**	**0.034**

## Discussion

In this study, the overall utilization of preconception care among HIV-positive women of reproductive age was 32%. This finding is lower than that reported in Nepal, where the utilization of preconception care was 51.0%^12^. The difference may be explained by stronger integration of reproductive health counseling services in Nepal, where a high proportion of women reported discussions with family planning providers, HIV care providers, and obstetric/gynecologic specialists regarding preconception care. In contrast, only 26.8% of women in the present study reported having discussions with healthcare providers about preconception care, indicating weak provider–client communication. This may reflect limited integration of preconception care into routine HIV services, weak implementation of guidelines, and differences in health system organization.

However, the finding of this study was higher than those reported in Mekelle City (18.2%), Hawassa (13.3%), Adet, East Gojjam (9.6%), and Debre Birhan (13.4%)^13–16^. This difference may be due to variation in study population and setting. The current study was conducted among HIV-positive women attending ART clinics who have regular contact with healthcare services and are more likely to receive reproductive health counseling. In contrast, the earlier studies were community-based, where access to structured counseling and health services is relatively limited.

This study may have overestimated preconception care utilization because routine HIV care services such as ART follow-up and general counseling were partly classified as preconception care components. This may have introduced misclassification bias. However, key components such as viral load assessment, family planning counseling, STI screening, nutritional counseling, and immunization were separately assessed to minimize this limitation.

In this study, 43% of HIV-positive women had good knowledge of preconception care. This finding is lower than that reported in Oromia Region, Ethiopia^17^, and lower than the pooled estimate from a national systematic review and meta-analysis in Ethiopia^18^. The difference may be related to variation in educational status, access to reproductive health information, and the level of integration of preconception care counseling into HIV services. Despite this, the finding indicates that a considerable proportion of women still lack adequate knowledge, highlighting the need for strengthened counseling and health education interventions.

The relatively low utilization of viral load assessment prior to conception (8.1%) observed in this study is lower than findings reported in other cross-sectional studies conducted in Ethiopia. For instance, a study conducted in West Shewa Zone reported that approximately 66% of women on ART had their viral load monitored as part of routine HIV care^19^. Similarly, findings from selected health facilities in Oromia Region showed that 70.4% of HIV-positive women received viral load testing during HIV care follow-up^20^. In addition, evidence from Addis Ababa indicated that viral load testing coverage among women receiving ART services exceeded 70%, reflecting better integration of laboratory monitoring within routine HIV care^21^. These differences may be attributed to variations in service integration, availability of laboratory infrastructure, and provider adherence to national HIV guidelines. The low utilization observed in the current study suggests gaps in linking preconception counseling with routine ART follow-up services.

In this study, only 4.1% of HIV-positive women utilized immunization services as part of preconception care, indicating very low uptake. This finding is lower than reports from other studies conducted in Ethiopia. For instance, a study in Addis Ababa reported that 58.6% of women living with HIV received recommended immunizations during HIV care services^21^. Similarly, findings from the Amhara Region showed that 46.2% of HIV-positive women accessed immunization services as part of reproductive health care^20^. In addition, a study from Southern Ethiopia reported 39.7% utilization of immunization-related preventive services among women attending HIV care^19^. The very low utilization observed in the present study may be due to poor integration of immunization services within preconception care, limited awareness of vaccine importance before pregnancy, and missed opportunities during routine ART follow-up visits.

According to the finding of this study, women who were able to read and write were more likely to utilize preconception care (PCC) services compared to women who were unable to read and write. Similarly, women with primary, secondary, and higher education levels were increasingly more likely to utilize PCC services than those with no formal education. This finding is consistent with studies conducted in West Shewa, Debre Birhan, Mekelle City, Adet (East Gojjam), and Southern Ethiopia^14–18^. Education improves awareness, access to health information, and understanding of reproductive health services, which in turn increases utilization of PCC^22–24^. Recent evidence also confirms that educational attainment is one of the strongest predictors of maternal and preconception health service use^25^.

Women’s monthly income was also significantly associated with PCC utilization. Women with higher income levels were more likely to utilize PCC services compared to those with lower income. This finding is supported by studies conducted in Debre Birhan and West Shewa Zone^15,16^. Socioeconomic status influences the ability to afford transportation and indirect costs related to accessing health services. A global review further highlights income inequality as a key barrier to maternal and preconception health service utilization in low-income settings^26^.

Occupational status was also significantly associated with PCC utilization. Private employees were less likely to utilize preconception care compared to government employees. This may be due to workload, limited flexibility, and difficulty accessing health services during working hours. Similar findings have been reported in studies showing that women in non-government employment have lower utilization of reproductive health services due to time and work constraints^27^.

Women with a history of pregnancy were more likely to utilize preconception care than those with no previous pregnancy. This may be because women with prior pregnancies have increased exposure to antenatal care and maternal health services, which improves awareness and utilization of PCC. Evidence from previous studies also shows that parity increases contact with health services and improves uptake of preconception and reproductive health interventions^28^.

### Strength and limitations

This study provides important evidence on preconception care utilization among HIV-positive women attending ART clinics and achieved a high response rate. However, the cross-sectional design limits causal inference. The findings may also be affected by recall bias and social desirability bias due to the use of interviewer-administered questionnaires. Since the study was facility-based, the results may not be generalizable to all HIV-positive women in the community. In addition, the inclusion of some routine HIV care services as components of preconception care may have led to an overestimation of preconception care utilization.

## Conclusion and recommendation

### Conclusion

Less than one-third of the ART clients utilize pre-conception care services. Educational status, occupation, monthly income, and history of pregnancy were significant predictors of preconception care utilization. The findings also revealed low utilization of important preconception care components, such as viral load assessment and immunization services. These results indicate gaps in the integration of preconception care within routine HIV services and highlight the need to strengthen reproductive health counseling and comprehensive preconception care delivery for women living with HIV.

### Recommendations

Based on the findings of the study the following recommendations are given for the concerned bodies.

Health institutions should strengthen on educating the importance of preconception care for all clients. Especially focuson less educational status, low monthly income and low and no history of pregnancy mother. Additionally, health institution arranges unit for pre-conception care service.

Nongovernmental organizations should support health institutions to increase preconception care utilization by availing appropriate logistics and building preconception care service room, Town, Zonal, and Regional Health Offices should give special attention to preconception care services like other maternal and child services.

Federal Ministry of Health, policy makers are advised to incorporate preconception counseling and care for HIV-infected women as a routine part of primary health care to improve the utilization of preconception care.

Researchers who interest in factors affecting PCC among ART should conduct a strong study design.

## Supplementary Information

Below is the link to the electronic supplementary material.

Supplementary Material 1

## Data Availability

Dataset may be made available from the corresponding author on reasonable request, subject to ethical approval and applicable institutional restrictions.

## Abbreviations

AIDS: Acquired immune deficiency virus
ANC: Antenatal care
ART: Anti-retroviral treatment
FP: Family planning
HCP: Health care provider
HIV: Human immune deficiency virus
IDSA: Infectious Disease Society of America
IUGR: Intra uterine growth restriction
MOH: Ministry of Health
MTCT: Mother-to-child transmission
PCC: Preconception counseling and care
PMTCT: Prevention of mother-to-child transmission
PrEP: Pre-exposure prophylaxis
STIs: Sexually transmitted infections
WHO: World Health Organization
