# Supplementary material for: Utilization of preconception care and associated factors among HIV-positive women of reproductive age attending ART clinics in government health institutions of Gamo Zone, Southern Ethiopia, 2021
Source: Sci Rep. 2026 Jun 24;16:22677. doi: 10.1038/s41598-026-58300-9 (PMC13385773; doi:10.1038/s41598-026-58300-9)
Supplement: Supplementary file 1 — Supplementary Material 1 [file 41598_2026_58300_MOESM1_ESM.pdf]

በደቡብ ብ/ብ/ሕ/ክልላዊ መንግሥት
አርባ ምንጭ ጤና ሳይንስ ኮሌጅ

South/N/N/P/Regional State
Arba Minch College of Health Sciences

Ref. No AMchs/[illegible]/7036
Date 02/07/13

To: **Birknesh Mereta** (Principal Investigator)

Subject: **Ethical Approval of a Research Proposal**

It is known that various research proposals has been passed through the Institutional Review Board of Arba Minch College of health Sciences, to this effect **Mrs. Birknesh Mereta** has submitted a research proposal titled **Knowledge, utilization of preconception care and associated factors among HIV positive reproductive age women attending ART clinics at Gamo zone government hospitals southern Ethiopia 2020/2021to** IRB office. The board has critically scrutinized the proposal for ethical issues and recommended the investigator to correct and incorporate essential elements. The investigator has incorporated all elements as required by the board. Therefore, the board approved the proposal through majority consensus of the board members on January 19, 2013e.c. The board acknowledges investigator for the concerted efforts he made to fulfill the recommendations of the board. However, since the board is bestowed to make follow-up of the research process. The investigator is informed with a copy of the approved letter to report any changes in the research procedure and submit an activity progress report to the IRB every month. This ethical clearance issued is valid for six (6) months and the institutional research review board may conduct an audit at any time.

With regards!

[illegible]

Vice Dean for Research and Community Service

Arba Minch College of Health Sciences

CC:

- Research and Community Service Directorate
